# Stakeholders perspectives on the key components of community-based interventions coordinating care in dementia: a qualitative systematic review

**DOI:** 10.1186/s12913-017-2725-y

**Published:** 2017-11-22

**Authors:** Amy Backhouse, David A. Richards, Rose McCabe, Ross Watkins, Chris Dickens

**Affiliations:** 1 University of Exeter Medical School, College House, St Luke’s Campus, Heavitree Road, Exeter, EX1 2LU UK; 2 National Institute for Health Research (NIHR) South West Peninsula Collaboration for Leadership in Applied Health Research and Care (CLAHRC), Exeter, UK

**Keywords:** Dementia, Health services, Dementia care coordination, Case management, Systematic review, Qualitative research, Collaborative care, Community interventions

## Abstract

**Background:**

Interventions aiming to coordinate services for the community-based dementia population vary in components, organisation and implementation. In this review we aimed to investigate the views of stakeholders on the key components of community-based interventions coordinating care in dementia.

**Methods:**

We searched four databases from inception to June 2015; Medline, The Cochrane Library, EMBASE and PsycINFO, this was aided by a search of four grey literature databases, and backward and forward citation tracking of included papers. Title and abstract screening was followed by a full text screen by two independent reviewers, and quality was assessed using the CASP appraisal tool. We then conducted thematic synthesis on extracted data.

**Results:**

A total of seven papers from five independent studies were included in the review, and encompassed the views of over 100 participants from three countries. Through thematic synthesis we identified 32 initial codes that were grouped into 5 second-order themes: (1) case manager had four associated codes and described preferences for the case manager personal and professional attributes, including a sound knowledge in dementia and availability of local services; (2) communication had five associated codes and emphasized the importance stakeholders placed on multichannel communication with service users, as well as between multidisciplinary teams and across organisations; (3) intervention had 11 associated codes which focused primarily on the practicalities of implementation such as the contact type and frequency between case managers and service users, and the importance of case manager training and service evaluation; (4) resources had five associated codes which outlined stakeholder views on the required resources for coordinating interventions and potential overlap with existing resources, as well as arising issues when available resources do not meet those required for successful implementation; and (5) support had seven associated codes that reflect the importance that was placed on the support network around the case manager and the investment of professionals involved directly in care as well as the wider professional network.

**Conclusion:**

The synthesis of relevant qualitative studies has shown how various stakeholder groups considered dementia care coordination interventions to be acceptable, useful and appropriate for dementia care, and have clear preferences for components, implementation methods and settings of these interventions. By incorporating stakeholders’ perspectives and preferences when planning and developing coordinating interventions we may increase the likelihood of successful implementation and patient benefits.

## Background

### Rationale

Health and social care services for dementia are currently fragmented and discordant. These issues have driven the development of policies and objectives such as the Challenge on Dementia 2020 [1] that focuses on delivering major improvements in dementia by advancing health and social care, creating dementia friendly communities and driving forward dementia research. Interventions that aim to coordinate care in dementia have been extensively researched such as case management and collaborative care interventions. These organisational interventions aim to coordinate care for individuals living at home in the community by assigning one service coordinator, usually a health or social care worker, who becomes responsible for all aspects of care. The service coordinator is most commonly known as the case manager (CM) and aims to manage the care of service users through a collaborative process of planning, facilitation and coordination [2]. Research has shown dementia care coordination interventions can improve health and wellbeing of individuals with dementia and their carers, and are seen as a beneficial tool in healthcare.

The results of a number of systematic reviews of community-based coordinating interventions have been mixed [2–7]. Pimouguet et al. [6] and Tam-Tham et al. [7] reviewed randomised controlled trials (RCTs) of dementia care coordination interventions and found it reduced the risk of institutionalisation, but reported no further impact on other health outcome measures. However, Somme et al. [5] reviewed RCTs and reported significant effects of these interventions on improving health outcomes for individuals living at home with dementia as well as reducing resource utilisation (i.e. hospital admission and institutionalisation). These data highlight the grey and uncertain landscape of evidence around coordinating interventions in dementia. This could relate to the heterogeneity in models of dementia care coordination interventions reported in the literature in terms of the target population, the components of the intervention, implementation and context, all of which reflect the diversity and complexity of these interventions. Importantly, although community-based dementia care coordination interventions can improve some health outcomes, it remains unclear as to how these interventions work and what components are important for improving outcomes.

Understanding the key components of such complex interventions and their effects is likely to be necessary to improve and refine the interventions. In order to address this, we conducted a review of the qualitative literature to explore the perspectives of stakeholders with respect to active components and mechanisms of effective community-based dementia care coordination interventions.

### Objectives

To synthesise the literature reporting the experiences, perceptions and views of stakeholders on community-based interventions coordinating care in dementia and their components.

## Methods

### Protocol and registration

We registered the review protocol with PROSPERO (registration: CRD42015024618), and it is published in BioMed Central Systematic Reviews [8] in accordance with the criteria in the Preferred Reporting Items for Systematic Reviews and Meta-Analyses (PRISMA) statement for systematic reviews [9].

### Eligibility criteria

#### Types of studies

We included studies that collected qualitative data on the experiences, perceptions and views of relevant stakeholders on dementia care coordination interventions. Study designs consisted of pure qualitative designs, mixed method designs and qualitative work embedded within quantitative studies.

#### Types of participants

Studies that involved relevant stakeholder groups including individuals with a dementia living in the community and their informal caregivers, CMs, and health and social care professionals.

#### Types of intervention

We defined dementia care coordination interventions as interventions that focused predominantly on planning, facilitating and coordinating care through proactive follow-ups, and delivered by a specified professional in a supporting role for provision and management of care. We included studies of coordinating interventions that were aimed at individuals with a diagnosis of dementia of any type who were living at home, with no restrictions on age, gender or comorbidities.

#### Setting

Interventions that were based in the community working with individuals still living at home. We excluded studies based in hospitals or nursing and residential homes.

#### Types of outcome measures

Qualitative data relating to the experiences, perceptions and views of relevant stakeholder groups on community-based interventions coordinating care in dementia.

#### Date, language and location

We placed no restrictions on date, language or study location.

### Information sources

#### Electronic searches

The following electronic databases from date of inception to June 2015, with the search syntax being modified appropriately for the individual database: MEDLINE (OvidSP), The Cochrane Library, EMBASE and PsycINFO.

#### Additional resources

A further four electronic databases were searched for grey literature; the Health Management Information Consortium (HMIC), Social Policy and Practice (SPP), ProQuest and the International Clinical Trials Registry Platform (ICTRP). We also conducted backward and forward citation searches on all included studies, and any relevant systematic reviews identified in the screening process.

### Search

A comprehensive search strategy was developed and deployed which used a combination of controlled vocabulary specific to the individual database (e.g. MEDLINE Medical Subject Headings (MeSH terms)) and free text terms. The master search strategy deployed in MEDLINE (OvidSP) can be found in Appendix 1.

### Study selection

#### Data management

All references were managed in EndNote X7.0.2. Titles and abstracts of studies identified in the initial search were imported into EndNote, followed by full texts of papers for further screening. Duplicates were removed automatically by EndNote, and assisted by a subsequent hand search.

#### Screening

Titles and abstracts were screened independently by two reviewers (AB, RW) against the inclusion criteria. Two reviewers then screened full texts of potentially relevant papers, with any screening disagreements being resolved through discussion or referral to a third reviewer if necessary.

### Data extraction

We used a bespoke data extraction sheet in Microsoft Office Excel software which was piloted by one reviewer (AB) on three qualitative studies and extracted data on study design, participants and quality. Nvivo v10 software was then used to extract and manage the findings from the qualitative data. We extracted qualitative data predominantly from ‘results’/‘findings’ sections of study reports, and additional relevant text under the ‘discussion’ sections.

### Risk of bias

We assessed study quality using the Critical Appraisal Skills Programme (CASP) qualitative research appraisal tool [10]. The checklist includes 10 questions covering rigour, research methods, relevance and research integrity. Quality assessment of all included studies was conducted by two independent reviewers (AB, RW), and disagreement was resolved through discussion. Although the appropriateness of excluding qualitative studies based on quality has been questioned [11], we used the assessment to comment on the quality of included studies across the ten CASP [10] questions.

### Method of analysis

We broadly followed the first two steps suggested by Thomas and Harden [12] for thematic synthesis. We began the analysis process with reading and re-reading the studies to develop an initial bank of codes that represented various concepts within the data. The text under the ‘results’/‘findings’ sections of each paper was extracted into Nvivo10 and underwent line by line coding. In this stage we assigned at least one code to each line of text. This process allows for translation of concepts across studies, and as synthesis progressed the code bank developed through adding, merging and altering codes as they emerged from the data. To ensure saturation of data, the discussion sections of each paper were searched for additional material and relevant data was coded.

For the second stage of thematic synthesis [12] we looked at the similarities and differences in the codes, and then grouped them based on the similarity in concepts and issues present in codes. This process created a hierarchical structure, with grouped codes forming descriptive, second-order themes.

## Results

### Study selection

**Fig. 1:**
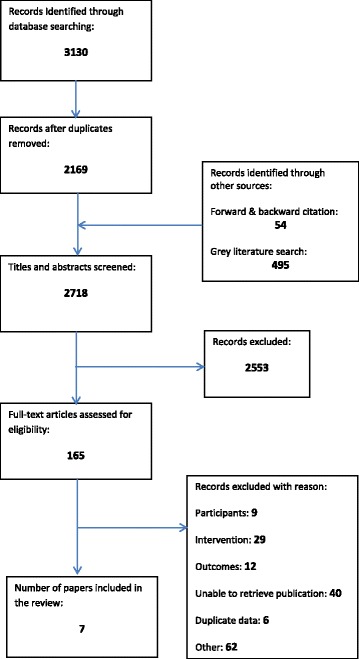
PRISMA flow diagram of study selection

We retrieved 2718 citations and included a total of seven [13–19] papers that came from five independent studies. A full report of the selection process can be seen in the PRISMA diagram in Fig. 1.

Three of the papers [13–15] included in the review were part of an HTA feasibility trial called CAREDEM. This meant the three separate papers were reporting on the same data, which was from a nested qualitative study within the feasibility trial. One paper [13] presented the sole findings of the embedded qualitative study and related findings to existing theory; another paper [14] presented a summary of the qualitative findings alongside those of the trial itself; and the final paper was the full HTA report [15] of the trial inclusive of the full qualitative results.

### Study characteristics

Table 1 shows a summary of the five studies (seven papers) included in the qualitative synthesis which were published between 2007 and 2014 [13–19].

**Table 1:** Summary of characteristics of included papers

Study ID	Country	Intervention	Design	Typology	Analytic Approach	Samples Size	Stakeholder Group
Iliffe 2014a [14] (Bamford 2014 [13]; Iliffe 2014b [15])	UK	Case management	Mixed methods study design	Process evaluation	Framework analysis	49	Person with dementia (6), carer (10), case manager (9), case manager mentor(4), research team members (2), GPs (6), administrative practice staff (5), community mental health team (2), voluntary sector workers (3), commissioners/ funders (2)
Gladman 2007 [16]	UK	Dementia support service	Qualitative study design	Service evaluation	Framework analysis	NR	GPs in the locality (6), old age psychiatrist (1), NHS patient advocates (NR), the team manager (1), representatives of the Carer’s Federation (NR), representative of Alzheimer’s Society (1), carers of service users (15)
Kosteniuk 2014 [17]	CANADA	Collaborative Care	Qualitative study design	Exploratory qualitative study	Thematic analysis	15	Family physicians (15)
Minkman 2009 [18]	NETHERLANDS	Case management	Qualitative study design	Multiple case study	Thematic analysis	16	Programme managers (8), case managers (8)
Van Mierlo 2014 [19]	NETHERLANDS	Case management	Multiple case study design	Process evaluation	Content analysis	22	Case managers (2), project leaders and care coordinators of care organisations (5), GPs (2), health insurance company representatives (2), mental health service representatives (2), programme coordinators of day care service (2), Alzheimer’s Netherlands representatives (3), municipalities stakeholders (3), informal caregiver support organisation representative (1)

#### Study participants

One study did not report the study sample size [16], and data from the CAREDEM study was reported across three papers [13–15]. There were over 100 participants across included studies, with one study [16] missing full data on all participants. The studies provide the perspectives of a range of relevant stakeholder groups with extensive experience in dementia care, which included General Practitioners (GPs) (29), carers of individuals with dementia (25), case managers (19), team/programme managers (9), voluntary sector workers (8+), persons with dementia (6), administrative practice staff (5), project leads and care coordinators of care organisations (5), case manager mentors (4), mental health services representatives (4), municipalities stakeholders (3), commissioners/funders (2), health insurance company representatives (2), programme coordinators of day care services (2), research team members (2) and old age psychiatrists (1).

#### Settings

Two [13–16] of the studies were based in the UK. Of the remaining three, two studies were based in the Netherlands [18, 19] and one in Canada [17].

#### Intervention

There were a number of different terms used to describe community-based interventions coordinating care in dementia. The majority of studies [13–15, 17, 18] referred to the intervention as case management; one study used the term collaborative care [17] and one dementia support service [12]. All of the interventions had a single coordinating individual responsible for planning, facilitating and coordinating care.

### Risk of bias

A summary of results from the CASP [10] quality appraisal can be found in Table 2, and full results to the ten CASP questions can be found in Appendix 2. All of the included studies had clear research questions, using appropriate methodology and design to address the question, with all but one study [16] using appropriate recruitment. Data collection was adequately described in all studies, and all had a clear statement of findings. However, in all studies it was also unclear as to whether the relationship between the researcher and participant had been adequately considered, question six of the CASP appraisal tool.

**Table 2:** Results of the CASP quality appraisal and classification of papers

Study ID	Number of questioned answered YES	Number of questioned answered CAN’T TELL	Number of questioned answered NO
Iliffe 2014a (Bamford 2014; Iliffe 2014b)	8	1	0
Gladman 2007 [16]	7	2	0
Kosteniuk 2014 [17]	7	2	0
Minkman 2009 [18]	6	3	0
Van Mierlo 2014 [19]	8	1	0

### Synthesis of results

We identified 32 codes and 5 second-order themes. The 5 seconds-order themes were; case manager, communication, intervention, resources and support. These 5 second-order themes are discussed in relation to their grouped codes:

#### Case manager

The case manager theme developed from four of the 32 codes; interpersonal skills, knowledge, professional background and training. Stakeholders across all studies felt aspects relating to the CMs personal and professional attributes were central to the success of the interventions [13–19]. Strong interpersonal skills, such as kindness and empathy [13–16] were important to service users, and thought to help the development of the therapeutic relationship, particularly with sensitive discussions around dementia and care [13–15].

Knowledge of dementia and knowledge of local services were the two central topics in CM knowledge data and primarily came from professional stakeholders [13–15, 17–19]. A core understanding of dementia was seen as important for the CMs to enable service users to manage the practicalities and uncertainties of the illness, as well as aiding with signposting duties [13–15, 18]. Some healthcare professionals felt CMs knowledge base was more important than their professional background [13–15, 19]. Training was one route to build on knowledge. Many professional stakeholders talked about the importance of providing early, role specific training for CMs [13–15, 17]. They referred to inductions to build on existing skills, using assessments to identify educational needs.

All studies [13–19] included data on the professional background of the CM, with the most commonly discussed backgrounds being nursing and social work. The majority of service users reported no clear preference for CM professional background. Nurses were seen to be more familiar to service users, they could offer more direct links to the GP and had the potential to address comorbidities. However, some professional stakeholders felt a primary care based nurse may medicalise the intervention, and may also lack comprehensive knowledge of local community services. Social workers on the other hand were seen as having a good knowledge of community services, and strong links to formal or paid services such as homecare or domiciliary care services. However, professional stakeholders suggested social workers might be more used to working in crisis situations and with larger scale, more complex needs, therefore may overlook smaller day-to-day needs [13–15].

#### Communication

Communication was a strong theme running throughout the data [13–19], and incorporated five of the 32 codes; role understanding, goals and aims, multi-disciplinary team, multiple organisations and research involvement. Professional stakeholders in some studies felt there was confusion over the scope and nature of the CM role [13–16, 19], suggesting an initial lack of a clear outline of the interventions led to uncertainties around the boundaries of the role and inconsistent implementation of the intervention. They felt a more precise outline would facilitate investment in the intervention, and improve collaboration and communication across partners [13–15, 19]. Despite inconsistencies across stakeholder groups in the overarching goals and aims of dementia care coordination interventions, many were in agreement that it should achieve a holistic and person-centred approach to dementia care [13–15, 18].

Communication in the form of feedback and meetings, alongside shared learning and resources, was seen by some respondents to facilitate a more efficient multidisciplinary team that could work collaboratively across primary, secondary and tertiary care [16–19]. By involving local services, dementia care coordination interventions could increase appropriate referrals, share knowledge and expertise, and form a broad dementia network. Professional stakeholders from one study stated this component broadens available information and promotes health and social care integration [19]. One study had substantial data relating to the involvement of a research team in studies of dementia care coordination interventions [13–15], who were seen as responsible for defining roles and supporting implementation through facilitating communication and collaboration.

#### Intervention

The intervention theme representing the practicalities of implementation incorporated 11 of the 32 codes; contact frequency, contact type, CM tasks, CM base, evaluation, identifying needs, illness trajectory, outcome measures, proactive, therapeutic relationship and workload. Many stakeholders felt contact type and frequency was dependent on the service user’s situation [13–15, 17, 18], a view that follows the person-centred ethos of dementia care coordination interventions. Service users preferred face-to-face contact, and although professionals saw the benefits of this, they highlighted the difficulty of time constraints and suggested telephone contact was a good substitute for maintaining regular contact.

Respondents felt that the initial stages of dementia care coordination should accommodate regular face-to-face contact in order to conduct a comprehensive assessment and develop the therapeutic relationship [13–16, 18, 19] described as a warm and trusting relationship between the CM, individual with dementia and the informal caregiver [17]. This relationship was important to many service users. Professional stakeholders felt identification of needs should be conducted through regular assessment by a trained CM, however this was prohibited by time constraints and the compromised ability of services users to identify their own needs [13–16]. Time constraints of CMs were a common barrier to implementation and dependent on workload which was associated with the CM caseload [13–15, 18, 19]. One study suggested the optimum number of cases per CM should be 50 [18].

There was a general consensus that dementia care coordination interventions should be offered at the point of diagnosis [13–18]. Many of these interventions aimed to reduce unplanned hospital admissions and institutionalisation, although this was seen by some as the wrong focus of the interventions since inevitably it will be the most appropriate action for the individual with dementia. At this point, respondents reported that the CM should play a role in facilitating the transition to a care home [13–15]. Stakeholders also drew attention to the importance of the health and wellbeing of the caregiver, with emphasis on measuring health-related outcomes of both parties in the dyad [13–16, 18].

A number of tasks were highlighted by professional stakeholders as relevant to the CM role [13–19]. Many of these were practical activities including; assessments, signposting, referrals, developing and implementing care plans, maintaining communication across services and partners involved. Professionals in one study also highlighted the importance of administrative duties including; recording visits, making field notes and updating patient record systems [13–15]. Evaluation was important to healthcare professionals who felt that there should be evidence of effectiveness and a clear trail of activity. This would allow for implementation issues to be identified early and corrected, and was also seen to facilitate collaboration and support for CMs [13–16].

The base of the CM was a point of contention between varying professional groups [13–16, 18, 19]. Many professionals felt the GP Practice was the most appropriate place for the CM as it was familiar to service users and could provide more direct links to the GP and other specialists. The practice was also seen to facilitate the uptake and support of dementia care coordination interventions and could promote team based working [13–15]. However, some professionals felt the intervention became ‘medicalised’ in this environment, which led to increased time pressures [13–15] and a community base could allow service users a break from more clinical health settings. However, difficulties were identified in the unfamiliarity of community services and the barriers of integrating with primary care [13–15].

#### Resources

Stakeholders across all studies discussed the resources required for successful implementation of dementia care coordination interventions, and the consequences when there is a lack of available resources [13–19]. The resources theme encompassed 5 of the 32 codes; available resources, existing roles, existing services, releasing other resources and time constraints. Time constraint was a common theme in the data, and affected many of the practical components of the intervention, with CMs in a part-time or dual role seemed to indicate the most time pressures [13–16, 19].

Most studies presented data around the potential overlap with existing roles and services [13–15, 17–19]. Admiral Nursing, community mental health nurses and dementia advisors were mentioned as having the potential to duplicate tasks leading to wasted resources. This issue was linked back to the lack of clarity and understanding of the CM role [13–15, 19]. Some professional stakeholders outlined aspects such as continuity of care and proactive monitoring of service users as novel, and were not covered to the same extent by other roles or services [13–15]. The collaboration that these interventions encourage across services was also highlighted, and many professionals felt it allowed a greater flexibility of involvement in care [13–15, 17, 18].

Some healthcare professionals stated a failure in dementia care coordination interventions could be attributed to the mismatch in available resources and those required for effective implementation [13–15]. Ensuring resources were used in the most cost-effective and appropriate manner was important to professionals [13–15, 17, 18]. One key benefit highlighted was the impact on alleviating pressure on other health resources, specifically GP appointment time. Several GPs mentioned they were unable to provide the necessary time to each individual during consultations, but CMs were able to save appointment time [13–15, 17].

#### Support

Healthcare professionals across all studies talked about the importance of a supporting network around the CM, and how this can be strengthened through investment by all participating parties [13–19]. The support theme developed from seven of the 32 codes; supervision, CM investment, continuity of care, GP investment, service user investment, team investment and value.

The investment codes refer to the value that stakeholders placed on the intervention, and the acceptance and willingness to participate. It was important to service users that the CMs showed enthusiasm for the role and acted as an advocate [13–15], but likewise professionals emphasised the importance of service users willingness to participate fully in order to gain the benefits [13–16, 19]. The investment of the wider group of professionals, including the GP and the immediate team, was also essential for success. This investment could ease the embedding of the CM into existing structures and encourage a supportive, collaborative network [13–15].

A number of supportive activities were reported as being in place for the CM, including supervision or mentoring. The mentor, who was often a senior healthcare professional, often covered tasks including individual case reviews, CM training and education needs, and encouraging integration of CMs within relevant teams [13–15].

Continuity of care was one of the valued components, and providing a single point of contact that offered long-term support was seen to facilitate continuity in the care process [13–15]. In one study, professionals highlighted that continuity enabled care to develop with the changing needs of service users, which was thought to help improve outcomes including crisis prevention [13–15].

## Discussion

### Summary of evidence

In this review we mapped five broad themes relating to intervention components that stakeholders felt were required for successful implementation from five studies on the experiences, perceptions and views of stakeholders on coordinated care for dementia. These categories were case manager, communication, intervention, resources and support.

Stakeholders generally considered community-based dementia care coordination interventions to be acceptable, useful and applicable to dementia care. The 32 codes not only included practical components such as contact type and frequency, supervision, training and evaluation, but also components that were based around the approach to care including support and investment across stakeholder groups, development of a good therapeutic relationship between CMs and service users and the value placed on dementia care coordination interventions.

There were some differences in the service user and professional stakeholder groups on their views of coordinating interventions. A significant amount of data came from professionals and seemed to focus on the practical elements and logistics of coordinating care. There was less data available from the service users, but analysis shows there is more focus on the personal aspects including the character of the CM and the ability to form a good therapeutic relationship in the right setting.

The difference in quantity of data from professionals and service users may have implications in the interpretation of results and reflect the relative infancy of involvement of service users in health research, and service planning and evaluation [20]. There is increasing evidence that involving service users enhances the suitability and acceptability of interventions, and aids the retention of participants in trials [21]. Systematically seeking input from service users during developmental stages of interventions, and actively seeking feedback in evaluation stages should be a key dimension for future research.

Our analysis has shown that stakeholders agree on a set of practical and philosophical underpinnings of community-based coordinating interventions. There is consistency in the preferred personal and professional attributes of CMs, in that they should be warm and empathetic with the ability to develop a strong therapeutic relationship with a sound knowledge of dementia and available local services. There is an agreed set of tasks the CM should complete including assessments, care planning, signposting and referrals, which should be conducted in a proactive manner and an agreement in regular contact that includes face-to-face meetings with service users.

Findings suggest that the CM should participate in meetings with the immediate multi-disciplinary team as well as meetings with wider health and social care professional network. However, there is still uncertainty around the details of key individuals or organisations in the wider professional network and precisely how often contact will need to take place. This is likely to be dependent on individual case as well as geographically dependent on local services available.

The implementation of the CM, and the coordination service as a whole, needs to be supported by the investment from individuals at the core; the individual with dementia, informal caregiver and the CM, as well as investment from the wider professional network. As such these understandings can be used to shape the next iteration of dementia care coordination interventions in way that makes them more acceptable to all concerned.

Analysis also highlighted some characteristics that may be barriers or may be helpful in the implementation of a care coordination service. Professional stakeholders suggested the significance of training before beginning the CM position and also saw the importance of have a mentor for guidance and supervision. Additionally, the embedding of an evaluation element was seen as practical for assessing the effectiveness of the coordinating care service.

A number of characteristics were highlighted as a particular hindrance during implementation including the caseload of the CM. High caseloads were difficult to manage and were reported to cause reactive care in crisis situations rather than the proactive follow-up care the interventions intended. This was strongly linked to the time constraints of CMs, particularly seen in CMs working in primary care. Furthermore, a lack in available resources was seen as a hindrance to implementation, and referred again to time constraints as well as wider issues like funding.

### Strengths and limitations

Evidence for our review was systematically identified, critically appraised and synthesised using the outlined steps for thematic synthesis. The studies we included in the review were of high to moderate quality, so the findings and conclusions carry considerable weight.

An important strength of this review is its focus on qualitative data to address questions of preference, concerns and applicability in dementia care coordinating interventions. A number of reviews have been completed in this area, but have focused on the effectiveness of coordinating interventions [2–7]. However, Khanassov et al. [4] included some qualitative evidence in a mixed-studies systematic review that aimed to highlight barriers to implementation. In agreement with some of our findings, frequently reported barriers included confusion of roles within the service delivery and a lack in communication among the professionals involved. Khanassov et al. [4] also reported numerous issues developing from time constraints of CMs including a change from proactive to reactive care, an issue reflected in the results of this review.

Although some of the themes found were more specific to coordinating interventions, such as the case manager theme, others were consistent with existing qualitative evidence in dementia care. Hirakawa et al. [22] found that limited resources such as a lack of time and space for quality dementia care, and a lack of funds for hiring and training interdisciplinary staff were barriers to integrated dementia care. Poor communication with professionals has also been associated with dissatisfaction in care from service users, and thought to influence the level of trust in the therapeutic relationship [23].

Although all studies focused on coordinating interventions that were community-based, they were from different countries which could affect the generalisability of findings. It is therefore important to consider the global differences in the structure and funding of health and social care services. However, based on the World’s Bank classification system [24], all three countries are high income countries where the state plays a significant role in the running of the healthcare system. Therefore the countries have broadly comparable health services and any differences in health care provision are unlikely to affect what stakeholders perceive as important in coordinating dementia care.

### Implications

Clinical services should consider these intervention characteristics when developing and implementing dementia care coordination interventions to help improve the effectiveness and optimise patient outcomes. Evidence from implementation science suggests that more careful thought at the development and planning stage can lead to better embedding of interventions into practice and a greater chance of success. For example, the PARiHS framework argues the interplay between evidence, context and facilitation will dictate the success of the implementation [25], and therefore services should consider these factors from the qualitative evidence available in the early stages of intervention development. Similarly the Normalisation Process Theory (NPT) states four main components important for an intervention to become ‘normalised’ in practice; coherence, engagement, collective action and reflexive monitoring [26]. During the initial intervention development, NPT provides a framework for mapping the context of the intervention and defining the individuals and groups involved. Using stakeholders can address questions within the framework including identifying the concerns of the individuals and groups, and determining whether the proposed intervention will address these concerns.

Further research is needed to determine which of these preferred characteristics are most important to the effectiveness of the intervention. Future trials of community-based dementia care coordination need to include a detailed process evaluation to capture the important information surrounding issues of implementation. In addition, it is essential that these trials provide detail on the exact content of interventions so they can be replicated, evaluated and compared.

## Conclusions

This synthesis of relevant qualitative studies has shown how various stakeholder groups have clear preferences for components, implementation methods and settings for community-based interventions coordinating care in dementia. By adhering to these preferences when planning and developing models of interventions we may increase the likelihood of success and produce more consistent results.

## Appendix 1

### Master Search Strategy in MEDLINE (OvidSP)

Database: Ovid MEDLINE(R) In-Process & Other Non-Indexed Citations and Ovid MEDLINE(R) < 1946 to Present >.

Search Strategy:

--------------------------

1 exp. Dementia/.

2 dement*.mp.

3 alzheimer*.mp.

4 (presenile/ or senile.mp.) and dement*.mp. [mp = title, abstract, original title, name of substance word, subject heading word, keyword heading word, protocol supplementary concept word, rare disease supplementary concept word, unique identifier].

5 *Delirium, Dementia, Amnestic, Cognitive Disorders/.

6 *cognition disorders/ or *mild cognitive impairment/.

7 1 or 2 or 3 or 4 or 5 or 6.

8 Case Management/.

9 collaborative care.mp.

10 case manag*.ti,ab.

11 care manag*.ti,ab.

12 (care adj2 coordinat*).ti,ab.

13 (case adj2 coordinat*).ti,ab.

14 service coordinat*.ti,ab.

15 care consult*.ti,ab.

16 case consult*.ti,ab.

17 (care adj2 facilitat*).ti,ab.

18 shared care.ti,ab.

19 (coordinat* adj2 care).ti,ab.

20 admiral nursing.mp.

21 *disease management/.

22 8 or 9 or 10 or 11 or 12 or 13 or 14 or 15 or 16 or 17 or 18 or 19 or 20 or 21 (33785).

23 7 and 22.

**************************************************************

[mp = title, abstract, original title, name of substance word, subject heading word, keyword heading word, protocol supplementary concept word, rare disease supplementary concept word, unique identifier].

[ti,ab = title & abstract]

## Appendix 2

**Table 3:** CASP Qualitative checklist

Study ID (author date)	1. Was there a clear statement of the aims of the research? (Y/ Can’t tell/ N)	2. Is a qualitative methodology appropriate? (Y/ Can’t tell/ N)	3. Was the research design appropriate to address the aims of the research? (Y/ Can’t tell/ N)	4. Was the recruitment strategy appropriate to the aims of the research? (Y/Can’t tell/ N)	5. Was the data collected in a way that addressed the research issue? (Y/ Can’t tell/ N)	6. Has the relationship between the researcher and participants been adequately considered? (Y/ Can’t tell/ N)	7. Have ethical issues been taken into consideration? (Y/ Can’t tell/ N)	8. Was the analysis sufficiently rigorous? (Y/ Can’t tell/ N)	9. Is there a clear statement of findings? (Y/ Can’t tell/ N)	10. How valuable is the research?
Gladman et al. 2007 [16]	Y	Y	Y	Can’t tell	Y	Can’t tell	Y	Y	Y	A valuable service evaluation assessing quality of care and coditions of care, which will be useful for replication of service.
Iliffe et al. 2014a [14]	Y	Y	Y	Y	Y	Can’t tell	Y	Y	Y	Adds substantial value in trying to replicate a US trial in the UK, and contributes valuable, detailed findings from process evaluation.
Kosteniuk et al. 2014 [17]	Y	Y	Y	Y	Y	Can’t tell	Y	Can’t tell	Y	Research contributes valuable findings from GPs views on coordinating interventions, but is lacking in detail and confined to rural settings.
Minkman et al. 2009 [18]	Y	Y	Y	Y	Y	Can’t tell	Can’t tell	Can’t tell	Y	Research is valuable in comparing variations of case management programmes, but needs more detail in findings.
Van Mierlo et al. 2014 [19]	Y	Y	Y	Y	Y	Can’t tell	Y	Y	Y	Provides useful and novel insight into the barriers and facilitators to delivering coordinating interventions.

## Abbreviations

CM(s): Case manager(s)
GP(s): General Practitioner(s)
NIHR: National Institute for Health Research
PenCLAHRC: Collaboration for Leadership in Applied Health Research and Care South West Peninsula
RCT: Randomised controlled trial
